# Rapid Evolution of Manifold CRISPR Systems for Plant Genome Editing

**DOI:** 10.3389/fpls.2016.01683

**Published:** 2016-11-14

**Authors:** Levi Lowder, Aimee Malzahn, Yiping Qi

**Affiliations:** Department of Biology, East Carolina UniversityGreenville, NC, USA

**Keywords:** sequence specific nucleases, RNA-guided endonucleases, CRISPR, Cas9, Cpf1, plant genome editing

## Abstract

Advanced CRISPR-Cas9 based technologies first validated in mammalian cell systems are quickly being adapted for use in plants. These new technologies increase CRISPR-Cas9's utility and effectiveness by diversifying cellular capabilities through expression construct system evolution and enzyme orthogonality, as well as enhanced efficiency through delivery and expression mechanisms. Here, we review the current state of advanced CRISPR-Cas9 and Cpf1 capabilities in plants and cover the rapid evolution of these tools from first generation inducers of double strand breaks for basic genetic manipulations to second and third generation multiplexed systems with myriad functionalities, capabilities, and specialized applications. We offer perspective on how to utilize these tools for currently untested research endeavors and analyze strengths and weaknesses of novel CRISPR systems in plants. Advanced CRISPR functionalities and delivery options demonstrated in plants are primarily reviewed but new technologies just coming to the forefront of CRISPR development, or those on the horizon, are briefly discussed. Topics covered are focused on the expansion of expression and delivery capabilities for CRISPR-Cas9 components and broadening targeting range through orthogonal Cas9 and Cpf1 proteins.

## Introduction

Sequence specific nucleases (SSNs) are quickly being incorporated into new and broadening research and development programs. Functional genomics studies, genetic engineering pipelines, molecular breeding activities and many other areas of plant research are all being impacted (Voytas, 2013; Voytas and Gao, 2014; Osakabe and Osakabe, 2015; Barakate and Stephens, 2016; Ma et al., 2016; Weeks et al., 2016). Of the primary SSN classes, clustered regularly interspaced short palindromic repeat (CRISPR) based platforms have been the most widely used and adopted in recent years (Graham and Root, 2015; Schiml and Puchta, 2016). Due to widespread incorporation and facile implementation, CRISPR-Cas9 (CRISPR-associated protein 9) based genome editing approaches have undergone rapid expansion, development, and improvement (Kumar and Jain, 2015; Paul and Qi, 2016; Zhang, D. et al., 2016). Evolution of technological optimization and enhancement has increased the usefulness, efficiency and capabilities of CRISPR-based genome editing technology.

Within the context of genome editing, CRISPR-Cas9 systems function by introducing DNA double strand breaks (DSBs) at genomic loci *in vivo* (Jinek et al., 2012; Cong et al., 2013; Mali et al., 2013). Previous gene targeting platforms, such as zinc finger nucleases (ZFNs, Sander et al., 2011; Qi, 2015), meganucleases (Smith et al., 2006; Pâques and Duchateau, 2007), and transcriptional activator-like effector nucleases (TALENS) (Christian et al., 2010; Li et al., 2011; Miller et al., 2011) also induce DNA DSBs against targeted chromosomal sites. However, CRISPR-Cas9 systems differ from these earlier nuclease platforms as Cas9 nucleases are guided to specific DNA sequences by small customizable RNA molecules (gRNAs) that form functional complexes with Cas9 within host nuclei and load the entire complex onto cognate chromosomal target sites for DSB induction (Jinek et al., 2012). The practical and economic implications of this difference translate into less time, cost, and effort spent executing genome editing experiments. ZFNs, meganucleases and TALENs have their respective strengths and provide additional application flexibilities, but RNA-guided endonucleases (RGENs) such as CRISPR-Cas9 are generally more facile, have been more widely adopted in recent years and are currently being optimized at an increasingly rapid pace. One caveat to this however, is that CRISPR-Cas9 can be less specific and more prone to off-targeting than TALENs because CRISPR-Cas9 recognition sequences (~23 bp including the PAM) are shorter than TALENs (typically >28 bp).

Once DSBs have been catalyzed by Cas9-gRNA complexes two possible fates proceed depending on cell type, target site, and DNA repair machinery. The vast majority of DSBs are hastily repaired by non-homologous end joining (NHEJ) mechanisms (Bortesi and Fischer, 2015; Paul and Qi, 2016). NHEJ DNA repair produces various indel and substitution mutations at DSB sites and thus is commonly used to knock out genes by introducing frameshift mutations early in protein coding sequences. NHEJ can be used for many different kinds of mutagenic applications such as molecular breeding, mutant library formation and high-throughput mutational screening (Bassett et al., 2015; Belhaj et al., 2015; Barakate and Stephens, 2016). Although far less likely and more difficult to isolate, DSB formation can also lead to homology-directed repair (HDR) recombination events (Schiml et al., 2014; Schiml and Puchta, 2016). HDR events occur if a homologous chromosome, or some other homologous DNA donor, is available to serve as a repair template. HDR based repair holds the greatest potential for precise genome editing but currently suffers from very low efficiencies.

A hallmark feature of CRISPR-Cas9 genome editing systems is that target DNA is recognized by Watson-Crick base pairing through reversible binding of gRNAs to Cas9 nucleases. Separating gRNA facilitated target acquisition from nuclease activity is contrary to ZFN and TALEN function which recognize DNA target sequences directly using protein-DNA interactions. This restricts any custom built isoform of ZFNs or TALENs to a single target locus; targeting any further loci requires designing and constructing additional ZFNs or TALENs. RGENs however can target many different genomic target sequences simultaneously provided that multiple gRNAs are expressed for each target site (Cong et al., 2013). Thus, CRISPR based reagents have a unique advantage for multiplex genome editing. Multiplexing greatly expands genome editing capabilities as it allows more efficient generation of large chromosomal deletion mutations and facilitates CRISPR based epigenetic genome modification (Paul and Qi, 2016; Puchta, 2016). Moreover, robust and easy to use molecular “toolkits” are available to streamline the assembly and expression of multiplexed gRNAs and support many different Cas9 variants and downstream applications (Xing et al., 2014; Lowder et al., 2015; Ma et al., 2015; Wang, C. et al., 2015; Zhang, Z. et al., 2016).

All manner of expression and delivery systems have been developed to enhance Cas9 and gRNA function in desired cell and tissue types (Ma et al., 2016) and more are currently being developed at the time of this writing. The different expression and delivery options available for CRISPR based experiments is largely dependent on highly specific applications. Researchers should be aware of the many different CRISPR based technical options now available for plants and what may be quickly arriving on the horizon. Here, we review the technical scope of these options and developments by loosely classifying them for easy reference and identification.

## Diversified expression of CRISPR system components

Initial mammalian and plant CRISPR-Cas9 vector expression systems were largely based on co-expression of one plasmid carrying Cas9 nuclease and a separate plasmid expressing gRNA. These types of vector systems are valuable for quick initial testing of simple gRNA/Cas9 functionality in highly specialized and transient expression systems. However, limitations of early vector systems became immediately apparent with the added expression complexity of gRNA multiplexing. Transient expression experiments could be useful for testing the efficiency of the constructs, even in the plant species that cannot be stably regenerated from protoplasts. However, transient expression systems may not be suitable for functional genetic studies that require high frequency inheritable genome editing. Thus, the development of a diversified set of plant CRISPR-Cas9 expression systems was undertaken to meet the needs of different plant research applications.

**Figure 1:**
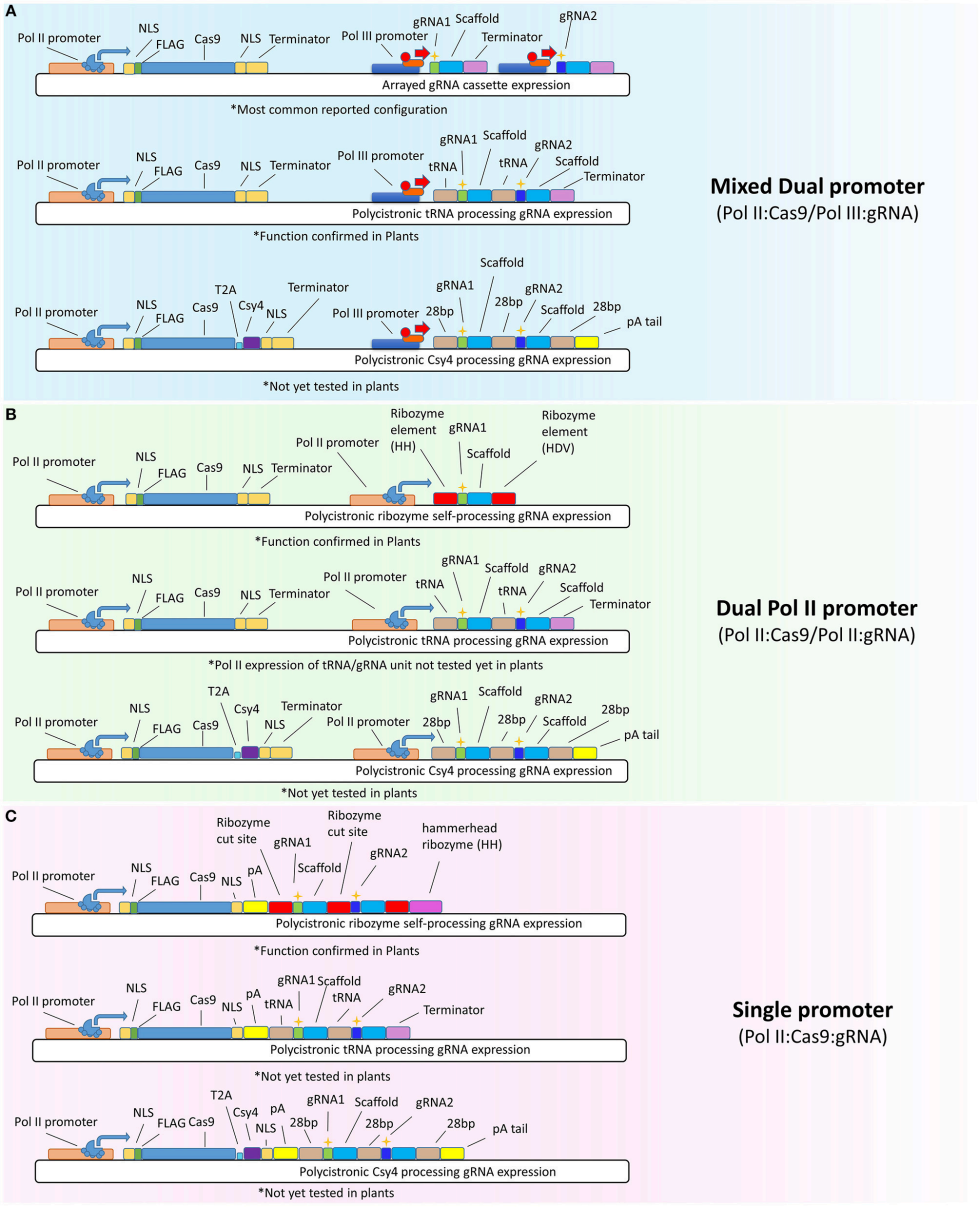
**Diversified plant CRISPR-Cas9 expression systems. (A)** The most common expression system is the mixed dual promoter system (Pol II:Cas9/Pol III:gRNA) where Cas9 is expressed from RNA polymerase II **(Pol II)** based promoters and gRNAs are expressed from RNA polymerase III promoters (Pol III promoters—such as U6 and U3). Top vector shows the canonical arrayed gRNA cassette expression system which expresses each gRNA with its own Pol III promoter, gRNA spacer, scaffold, and terminator sequences. Cas9 is expressed from a Pol II promoter and typically has N and C terminal nuclear localization signals **(NLS)**, a **FLAG** tag for immunodetection, and a 3′ transcriptional terminator sequence. Middle vector shows the polycistronic tRNA processing gRNA expression system which expresses Cas9 using Pol II and gRNAs from a polycistronic Pol III driven transcript. tRNA encoding sequences act to process out RNA sequences they flank using endogenous tRNA processing ribonucleases. Bottom vector is the polycistronic Csy4 processing gRNA expression system that has been tested to function in mammalian cells, but not yet in plants. The Csy4 system utilizes the CRISPR type III ribonuclease, Csy4, to cleave the **(28 bp)** sequence which cuts out RNA sequences flanked by these sequence elements. A poly A tail **(pA tail)** is used to stabilize the 3′ RNA transcript sequence after processing. **(B)** The Dual (Pol II) promoter system (Pol II:Cas9/Pol II:gRNA) uses RNA polymerase II promoters to drive both Cas9 and gRNA expression. Different Pol II promoters can be used simultaneously to express the separate Cas9 or gRNA transcripts providing great flexibility and capacity for constitutive or inducible RNA expression. Top vector shows the polycistronic ribozyme self-processing gRNA expression system which processes out gRNAs from Pol II primary transcripts using the hammerhead **(HH)** and **(HDV)** ribozyme cleavage sequence elements. Middle shows the polycistronic tRNA processing gRNA expression system from **(A)** but adapted for the dual promoter system by Pol II promoter controlled transcription. Bottom shows the Csy4 processing gRNA expression system as above but under the control of two separate Pol II promoters. Note that **Csy4** ribonuclease must be expressed from the Cas9 transcript using the translational viral cleavage sequence **(T2A)** which allows for two functional polypeptides to be produced from a single transcript. **(C)** Shows the polycistronic ribozyme self-processing system, the tRNA processing and Csy4 processing gRNA expression systems under the the control of a single Pol II promoter. Note that the single promoter system is the most compact of all the systems. ^*^Experimentally validated function in plants is denoted below vector.

No matter the application or layout of any single expression system the end requirement of Cas9 based genome editing is the same: it requires that Cas9 and gRNA(s) be present in the host nucleus at sufficient concentration. If both elements are to be transcribed *in vivo* then some sort of transcriptional promoter will be required. Typically, Cas9 is expressed from an RNA polymerase II (Pol II) based promoter (Figure 1). The majority of studies published to date use some variant of the constitutively active promoters such as *Cauliflower mosaic virus* (CaMV) *35S* promoter or *Arabidopsis* Ubiquitin 10 (*AtUbi*) promoter to drive Cas9 expression in dicots. Maize or rice Ubiquitin *(ZmUbi*; *OsUbi*) promoters are most commonly used for constitutive Cas9 expression in monocots. Constitutive promoters such as *35S* and *Ubi* are useful for testing proof of concept studies or preliminary investigations but result in high levels of somatic cell mutation and mosaic T0 or T1 plants. When producing heritable transgenic lines, it is desirable to use various egg or germline specific promoters. This has been shown to be important for plants such as *Arabidopsis thaliana*, which is generally transformed using Agrobacterium mediated floral dip that targets ovule cells for transformation (Desfeux et al., 2000; Mao et al., 2015; Wang, Z. P. et al., 2015). Importantly, it was proposed that *CaMV 35S* constitutive promoter activity may be less effective in egg or single cell embryos (Wang, Z. P. et al., 2015).

gRNAs are typically expressed under small nuclear RNA promoters such as the U6 and U3 promoters. These small nuclear RNA promoters are constitutively transcribed by RNA polymerase III (Pol III) and require a specific 5′ nucleotide to initiate transcription (5′-Guanine for U6 or 5′-Adenine for U3) (Jiang et al., 2013; Li et al., 2013; Nekrasov et al., 2013; Shan et al., 2013; Lowder et al., 2015). U6 and U3 promoters work well to express gRNAs in plants but they are not ideal for certain gene targeting applications due to lack of spatiotemporal control and the requirement of extra nucleotide restrictions on 5′ ends of target sequences or mismatch nucleotide additions on gRNA sequences (Gao and Zhao, 2014; Xie et al., 2015; Yoshioka et al., 2015; Tang et al., 2016).

## Pol II:Cas9 and Pol III:gRNA—mixed dual promoter systems

Early reports of CRISPR-Cas9 function in plants generally utilized the canonical mixed dual promoter expression system; i.e., they expressed a heterologous Cas9 under an RNA Polymerase II promoter and a separate gRNA cassette driven by the RNA polymerase III U6 or U3 promoters (Jiang et al., 2013; Li et al., 2013; Nekrasov et al., 2013; Shan et al., 2013, Figure 1A). Li and colleagues utilized the first reported plant codon optimized Cas9 (pcoCas9) and observed mutation efficiencies below 6%. By varying coexpression levels of Cas9 and gRNA these authors concluded that gRNA expression was the limiting factor for maximal mutagenesis efficiencies in *Arabidopsis* protoplasts using *35S PPDK* to drive the transcription of Cas9 (Li et al., 2013). Nekrasov and colleagues utilized *35S* promoter to express human codon optimized Cas9 and observed 6.7% mutational frequencies in Agrobacterium infiltrated tobacco leaves (Nekrasov et al., 2013). Shan et al., showed up to 20% mutation frequency in rice protoplasts using a rice codon optimized Cas9 and a 2 X *35S* promoter (Shan et al., 2013). Reported mutation efficiencies of these initial studies were relatively low and may correspond to early renditions of plant Cas9 codon optimization, decreased co-expression transformation efficiency, poor Cas9 expression stability, suboptimal gRNA expression, cell system limitations, or suboptimal target sites (Bortesi and Fischer, 2015; Yan et al., 2015; Ma et al., 2016).

Multiplexing gRNA expression in the mixed dual promoter system is accomplished by stacking or tiling multiple gRNA expression cassettes together in series (Li et al., 2013; Lowder et al., 2015; Ma et al., 2015, Figure 1A, top panel). This approach requires each gRNA cassette to harbor a small RNA promoter (U6 or U3), gRNA spacer targeting sequence, gRNA scaffold sequence and a 3′ terminator element. Cloning and assembly of multiplexed gRNA cassettes was once laborious and time consuming, but has recently been drastically simplified and streamlined by toolkits available at public repositories (Lowder et al., 2015).

Assembled gRNA cassettes are each generally 300–600 nucleotides in length. Due to their size, stacking cassettes for multiplex expression can quickly create large heterologous expression vectors that are potentially prohibitive; especially when size-restrictive viral vectors are used for delivery (Baltes et al., 2014). Moreover, *Agrobacterium* based T-DNA transgene insertion is less restricted by large transgene sizes but it is unknown whether stacking multiple small RNA promoters/gRNA cassettes together for random insertion into plant chromosomes has any detrimental transcriptional silencing effects. It has been well established for some time that both cis and trans transcriptional gene silencing increases with the number of gene copies at single loci (Vaucheret and Fagard, 2001). Thus, gene stacking of multiple gRNA cassettes together at a single locus may lead to increased susceptibility for transgene silencing. Silencing of T-DNA insertions imposed by gRNA cassette stacking could also lead to silencing of the closely arranged Cas9 gene given that silencing is usually induced by formation of heterochromatin and DNA hypermethylation at regions proximal to transgene insertion sites. Supporting this notion, Xie et al. (2015) obtained high levels of mutation frequencies by expressing multiplexed gRNAs from the polycistronic transfer RNA (tRNA) processing system under a single U3 Pol III promoter (Figure 1A, middle panel, Xie et al., 2015). Mutational frequencies from this study reached up to 100%. However, comparing mutational frequencies between earlier studies using stacked Pol III cassettes (9–70%) with polycistronic expression results is cautioned against at this time as mutational frequencies are not always comparable between studies nor are they always indicative of expression stability or effectiveness. Polycistronic Csy4 processing gRNA expression system was shown to function in mammalian cells (Tsai et al., 2014). The Csy4 system utilizes the CRISPR type III ribonuclease, Csy4, to cleave the 28 bp sequences that flank the gRNAs (Haurwitz et al., 2010). When compared with the tRNA system, it will be interesting to see whether a mixed dual promoter system based on Csy4 will function efficiently in plants (Figure 1A, bottom panel).

Constitutive expression of gRNAs under small RNA promoters (U3 & U6) is efficient enough for many CRISPR based applications. However, for applications that require more control over mutation heritability, involve potentially toxic mutations or warrant spatial or temporal control over genome editing, then tissue-specific or inducible gRNA expression is preferable. Multiple groups have tested germline-specific promoters for driving Cas9 expression in order to obtain high frequency germinal editing in *Arabidopsis* (Hyun et al., 2015; Mao et al., 2015; Yan et al., 2015; Wang, Z. P. et al., 2015; Eid et al., 2016). These studies demonstrate that controlling Cas9 transcription alone while maintaining constitutively high gRNA expression is effective for generating heritable mutations in *Arabidopsis*. However, their studies did not directly observe how tightly controlled Cas9 is to relative cell types or if any unintended Cas9 expression occurred. The importance of this issue was recently highlighted by two studies showing unintended CRISPR-Cas9 genome editing in *C. elegans* and mammalian cells *in vivo* and *in vitro* (Shen et al., 2014; Dow et al., 2015). These animal studies constitutively expressed gRNAs from Pol III snRNA promoters and controlled Cas9 expression under tissue specific promoters but still observed off-tissue targeting and recommend caution from expressing gRNAs ubiquitously for tissue specific gene editing. Based on these reports, further plant studies will be needed to address Cas9 promiscuity under tissue-specific or inducible promoters while gRNA expression is constitutive.

Since the first reports of CRISPR function in plants, mixed dual promoter systems have become the overwhelming standard for CRISPR-Cas9 experiments. However, possible limitations of this strategy for multiplex applications has led to the very recent developments of different expression systems that modify either the gRNA promoter or condense multiple gene stacks to polycistronic genes.

## Pol II::Cas9 and Pol II::gRNA—dual Pol II promoter systems

The wide diversity and availability of constitutive, inducible and cell type specific Pol II promoters highlights the potential benefits of dual Pol II promoter systems where both Cas9 and gRNAs are expressed under Pol II promoters (Figure 1B). Dual Pol II promoter systems allow for enhanced control of CRISPR-Cas9. Expression of gRNAs from Pol II promoters requires mRNA processing of primary transcripts to form functional gRNA units. An earlier study utilized this expression strategy for genome editing in wheat cells but obtained low Cas9 mutagenesis activity, presumably due to low activity of unprocessed Pol II expressed gRNAs (Upadhyay et al., 2013). Proper processing of Pol II expressed primary RNA for mature gRNAs has been demonstrated by utilizing the hammerhead and HDV ribozyme RNA self-cleavage system in yeast (Gao and Zhao, 2014), *Arabidopsis* (Gao, Y. et al., 2015), and mammalian cells (Yoshioka et al., 2015) (Figure 1B, top panel). Alternatively, the Pol II expressed gRNA containing primary RNA can be processed by the Csy4 RNA cleavage system as demonstrated in mammalian cells (Nissim et al., 2014) (Figure 1B, bottom panel).

Controlling Cas9 expression while simultaneously regulating gRNA expression under a separate Pol II promoter has the potential to offer the most flexibility and control over spatiotemporal induction of genome editing events, especially if different sets of gRNAs are to be expressed at different times or in different tissues simultaneously or sequentially. Moreover, heterologous U3 and U6 promoters exhibit high rates of variable expression in non-model organisms and many crop plants where snRNA promoters have not been well studied or identified (Sun et al., 2015; Tang et al., 2016). Hence, the use of Pol II promoters to express gRNAs will give researchers more choices and confidence when applying CRISPR-Cas9 systems to non-model plants in which Pol II promoters are better studied and tested than Pol III promoters.

Combining the Dual Pol II promoter expression strategy with polycistronic gRNA expression offers great potential to provide the most diverse and robust CRISPR-Cas9 expression system. This strategy has not yet been carried out in plants, but has been used in transient mammalian cell culture systems utilizing the CRISPR type III Csy4 RNA cleavage nuclease (Nissim et al., 2014). tRNA polycistronic processing of gRNAs using RNase P and Z has not yet been reported under the control of a Pol II promoter. RNase P and RNase Z are highly conserved and ubiquitously distributed tRNA processing ribonucleases. RNase P and Z recognize tRNA stem-loop secondary structures specifically for cleavage and are not dependent on nearby sequence contexts. However, tRNAs are naturally expressed in eukaryotes from RNA Pol III promoters and therefore expression of functional tRNA polycistronic gRNAs from Pol II promoters is not yet known to function efficiently in plants. In the future, testing and quantifying *In planta* expression of multiplexed gRNAs with a single Pol II primary RNA transcript will be of general interest, whether it is based on ribozyme processing, Csy4 processing or tRNA processing (Figure 1B).

## Pol II Cas9:gRNA—single transcript unit systems

Recently, Yoshioka and colleagues developed a mono-promoter CRISPR-Cas9 system in which both Cas9 and gRNA are expressed as a single transcript by a single Pol II promoter (Yoshioka et al., 2015). In their design, they put an HH-gRNA-HDV cassette ahead of Cas9 by linking two components with an internal ribosome entry site (IRES). They found this single transcript system led to mutagenesis frequencies of ~5% in mammalian cells, which is about half the efficiency of dual Pol II promoter systems (Yoshioka et al., 2015). More recently, an effective single transcript unit (STU) system was reported for CRISPR-Cas9 expression in both dicot and monocot plant cells (Tang et al., 2016). High levels of CRISPR-Cas9 mutagenic frequencies (up to 100%) were achieved by this STU system (Tang et al., 2016). It fuses Cas9 expression and gRNA expression together into a single cohesive transcript by positioning Cas9 in front of gRNAs (contrary to the above-mentioned mammalian system) and taking advantage of the cis-acting hammerhead ribozyme (RZ) (Figure 1C). This approach may not match the ultimate flexibility and versatility of the dual Pol II promoter strategy (Figure 1B), but is more streamlined and elegant. The simplified nature of this expression strategy may offer higher expression capacity and is more amenable to viral vector based delivery of CRISPR-Cas9 reagents, which require compact nucleotide payloads (Baltes et al., 2014). Expression systems based on this strategy could be expanded to include polycistronic tRNA processing gRNA expression vectors and the Csy4 type III CRISPR RNA processing system.

## Delivery of CRISPR-Cas9 into plant cells

**Figure 2:**
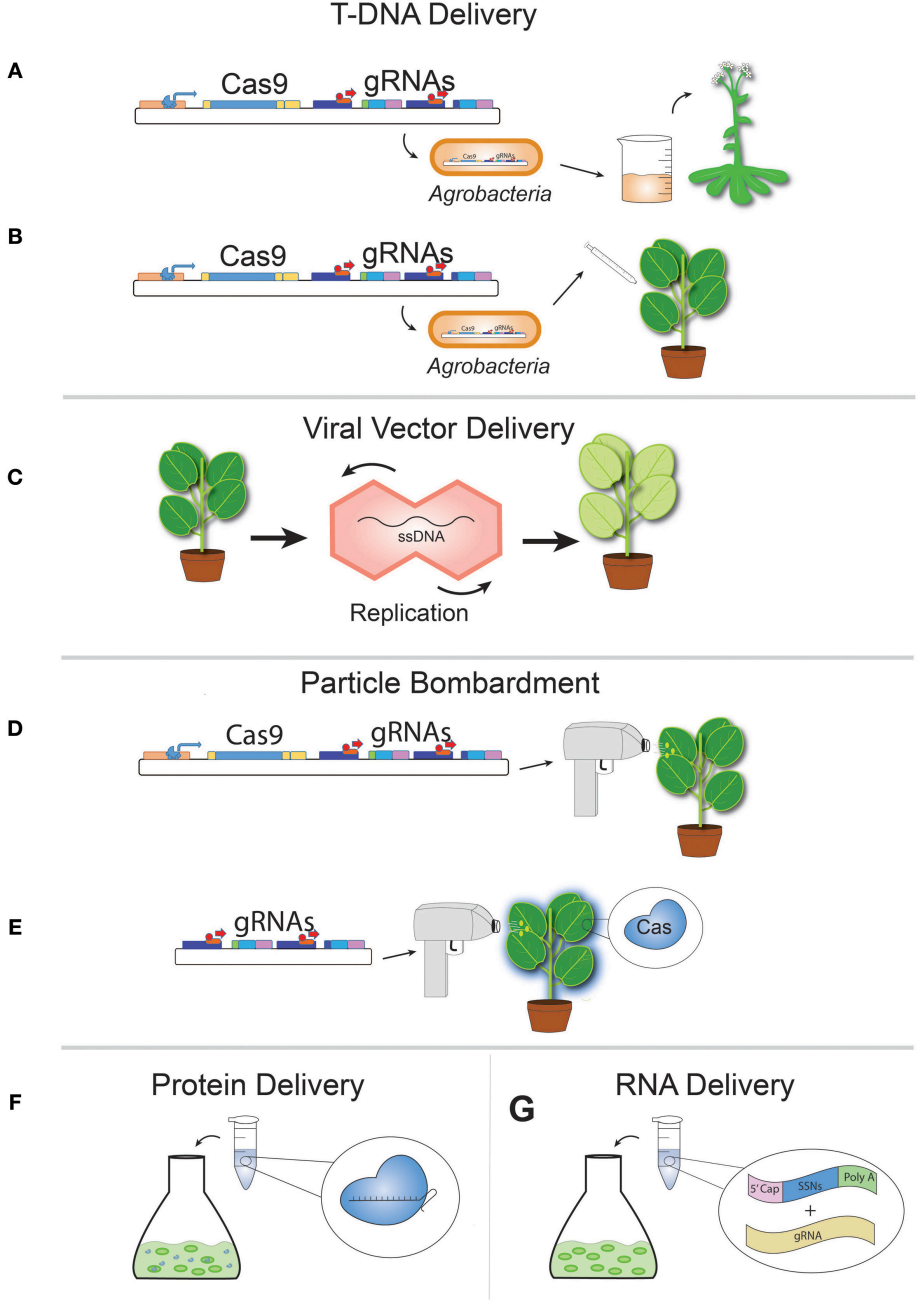
**Delivery of CRISPR reagents to plant cells and tissues. (A)** Floral dip transformation of *Arabidopsis* with transgenic T-DNA carrying *Agrobacteria*. **(B)** Transient inoculation of plant leaf tissue or calli with *Agrobacteria* harboring Cas9 and gRNA T-DNA. **(C)** Viral vector delivery causes a transiently transformed plant (at left) to develop systemic infection upon viral capsid replication after initial transformation of vector DNA. **(D)** Transient particle bombardment of plant leaf tissue using a gene gun with Cas9 and gRNA or **(E)** gRNAs only to stable Cas9-expressing transgenic plants. **(F)** Ribonucleoprotein (RNP) complex delivery directly to protoplasts using PEG transformation or **(G)** RNA delivery directly to protoplasts (shown here) using PEG transformation or calli using “gene gun” as in **(D)**.

There are many different options for delivering CRISPR-Cas9 reagents to plant cells (Figure 2). Reagents can be expressed from heterologous transgenes incorporated into plant cells as either DNA or RNA, or transported directly to nuclei as functional ribonucleotide protein complexes. Delivery systems vary based on plant species, research purposes, available expertise and equipment. Specific delivery options can also aid in the marketability of non-GM genome edited crops by circumventing restrictive regulatory burdens (Wolt et al., 2016).

## Agrobacterium mediated T-DNA delivery

Agrobacterium mediated T-DNA transformation is the predominant method for generating transgenic plants. In the past few decades, specific Agrobacterium transformation methods have been developed and optimized for different plant species (Wang, 2015). Not surprisingly, Agrobacterium mediated T-DNA transformation has emerged as the most widely used approach to deliver CRISRP-Cas9 expression DNA cassettes into plant cells. For example, CRISPR-Cas9 mediated genome editing in the model plant *Arabidopsis* is exclusively carried out by floral dip Agrobacterium mediated transformation (Figure 2A, Clough and Bent, 1998; Feng et al., 2013; Jiang et al., 2013; Li et al., 2013). Genome editing of tobacco plants begins with transformation of somatic cells by Agrobacterium tissue infiltration (Figure 2B) followed by regeneration of T0 plants using standard tissue culture techniques (Nekrasov et al., 2013; Gao, J. et al., 2015). The highly efficient Agrobacterium transformation of mature embryos in rice makes this system a popular and effective platform for CRISRP-Cas9 applications (Feng et al., 2013; Mao et al., 2013; Miao et al., 2013; Shan et al., 2013; Xie and Yang, 2013). For maize, CRISPR-Cas9 relies on Agrobacterium based transformation of immature embryos and appears to be effective in this important crop species (Svitashev et al., 2015; Char et al., 2016).

Plant transformation methods, especially Agrobacterium mediated transformation, will be a major limiting factor for adopting CRISPR-Cas9 technology to many other plant species. We anticipate revived interest in developing effective Agrobacterium mediated transformation methods in currently recalcitrant plants. A major advantage of genome editing is that desirable outcomes can often be achieved using transient expression of CRISRP-Cas9 systems. Hence, it is not necessary for CRISPR-Cas9 transgenes to integrate directly into plant genomes. This feature allows for diverse alternative approaches for delivering CRISPR-Cas9 reagents, which are discussed in detail below.

## Viral delivery

Plant RNA and DNA viruses have great potential for efficient reagent delivery to a wide diversity of plants (Figure 2C). Viral delivery systems have been used in mammalian and plant cell systems to deliver various genome editing reagents including ZFNs, TALENs and RGENs. Single stranded DNA geminivirus-based replicons were employed to effectively deliver ZFNs, TALENs, and CRISPR-Cas9 reagents to tobacco and increased gene targeting frequencies up to two orders of magnitude over *Agrobacterium* mediated T-DNA transformation (Baltes et al., 2014). Similarly, Yin et al., described their virus-based gRNA delivery system for CRISPR-Cas9 mediated genome editing or VIGE delivery method. VIGE utilizes the Cabbage Leaf Curl geminivirus to express gRNAs in stable Cas9 overexpressing lines of tobacco and was shown to be highly effective at inducing systemic infection and RGEN mediated mutagenesis (Yin et al., 2015). By delivering gRNA alone to Cas9 stable plants using VIGE, Yin et al., were able to compensate for the low nucleotide cargo capacity of the geminivirus vector. Tobacco Rattle virus was also used to effectively induce systemic plant infections of CRISPR-Cas9 encoding RNA viral genomes, leading to the generation of mutated T0 plants. Stable inheritance of mutations to T1 plants was observed although the authors of this study recommend that germinal transmission of mutations using tobacco rattle virus based delivery be further studied to enhance efficiency of stable mutant progeny regeneration (Ali et al., 2015). These studies provide great promise for delivery of RGENs to crop species as plants can be transiently infected relatively quickly and viral replication of reagents can spread to systemic infection of whole plants (Figure 2C). Compared to the laborious and highly technical process of protoplast transformation and biolistic transformation followed by plant tissue culture dependent regeneration of stable mutants, viral systems offer a more facile and efficient delivery option that may be more widely adaptable to a diversity of crop taxa. However, such systems are currently limited by low editing efficiency in germline cells.

## Plasmid delivery

Rather than being carried by Agrobacterium T-DNA, CRISPR-Cas9 cassettes can be delivered into plant cells by expression plasmids. This is typically achieved by PEG transformation of protoplasts or biolistic particle delivery using a gene gun (Figures 2D,E). Whether being integrated chromosomally or expressed episomally within the nucleus, CRISPR-Cas9 transgenes can be effectively expressed. Tailored for different purposes, both approaches are generally robust enough to achieve desired delivery of CRISPR-Cas9 reagents. The protoplast system is mainly used for rapid testing of CRISPR-Cas9 activity in plants cells and such assays have been routinely performed in *Arabidopsis* (Li et al., 2013; Tang et al., 2016), tobacco (Li et al., 2013; Tang et al., 2016), rice (Shan et al., 2013; Tang et al., 2016), and generally can be applied to almost any plant. In contrast, gene gun based biolistic delivery is primarily used to transform plant tissues or embryos for subsequent regeneration of stably edited plants. This technique has been successfully applied to major crops such as rice (Sun et al., 2016), maize (Svitashev et al., 2015), wheat (Zhang, Y. et al., 2016), and soybean (Li et al., 2015). Regeneration of plants from protoplasts and intact somatic cells can be challenging and time consuming. It is especially difficult to achieve germline editing in plants that are either resistant to many transformation methods or are laborious and expensive to work with. Improving regeneration based plasmid delivery approaches will become an important research priority to open up CRISPR-Cas9 delivery to these recalcitrant plants.

## Ribonucleotide protein complex delivery

Previously, ZFNs were successfully delivered as proteins into mammalian cells to mediate genome editing (Gaj et al., 2012). More recently, protein delivery of TALENs was demonstrated in tobacco protoplasts (Luo et al., 2015). Protein delivery of SSNs may have advantages for certain applications such as avoiding potential regulatory burdens put on transgenic crop improvement or genome editing of specialty crops that are propagated asexually. A disadvantage of CRISPR-Cas9 is that a pure protein delivery is impossible due to the requisite gRNA component. However, direct transfer of purified and preassembled Cas9-gRNA ribonucleoprotein complexes (RNPs) was demonstrated in human cells (Kim et al., 2014). A year later, the same group successfully applied this method to protoplasts of different plant species including *Arabidopsis*, tobacco, lettuce, and rice (Woo et al., 2015, Figure 2F). Currently, direct delivery of CRISPR reagents as protein complexes requires regeneration of plants from infused protoplasts, which is only efficient for a limited assortment of species (Woo et al., 2015; Ma et al., 2016).

## RNA delivery

A similar strategy carried out very recently utilizes the transfer of RNA encoding genome editing reagents directly into plant cells (Stoddard et al., 2016; Zhang, Y. et al., 2016, Figure 2G). *In vitro* mRNA transcripts of CRISPR-Cas9 and gRNA were co-bombarded successfully into wheat calli, although mutation frequencies using this approach were very low-1.1% (Zhang, Y. et al., 2016). Low mutagenesis frequencies using bombarded transient RNA appear to be caused by a short half-life of intracellular RNA stability. In another study, a pair of TALENs (Stoddard et al., 2016) were delivered by mRNA PEG transformation of tobacco protoplasts and mutational efficiencies were observed to be highly dependent on 5′ and 3′ untranslated regions (UTRs) of mRNA molecules. The authors in both studies note that reagent delivery by mRNA can induce genome editing without transgene insertion into host genomes (Stoddard et al., 2016; Zhang, Y. et al., 2016). This also may help skirt regulatory restrictions and decrease negative side effects associated with randomly inserted transgenes that can disrupt host genome structure and function. An additional benefit of purified RNP or RNA delivery of RGENs over more conventional DNA delivery is that RNPs and mRNA degrade quickly after mutagenesis. Transient reagent delivery that results in stable mutagenesis drastically reduces the probability of off-targeting that could negatively impact plant function, fertility or growth (Luo et al., 2015).

## Broadening targeting ranges with orthogonal CRISPR-Cas9 systems

The CRISPR-Cas9 system can be further broadened with the introduction and application of orthogonal CRISPR-Cas9 systems, such as RGENs from *Streptococcus thermophiles* (St), *Neisseria meningitidis* (Nm), and *Staphylococcus aureus* (Sa), which have been demonstrated previously to function in mammalian cells (Esvelt et al., 2013; Ran et al., 2015). Recently, StCas9 and SaCas9 mediated genome editing was shown in *Arabidopsis* (Steinert et al., 2015), and SaCas9 mediated genome editing was reported in tobacco and rice (Kaya et al., 2016). These orthogonal Cas9 systems use different Cas9 proteins, PAM requirements and gRNA scaffolds for target recognition, hence expanding the targeting sites defined by the most popular SpCas9 system. We note that the above-mentioned expression and delivery systems also apply to orthogonal CRISPR-Cas9 systems. Orthogonal Sp, St, Nm, and Sa Cas9 enzymes all generate blunt-ended DNA DSBs and produce relatively similar genome editing outcomes.

## Will CRISPR-Cpf1 futher boost plant genome editing?

**Figure 3:**
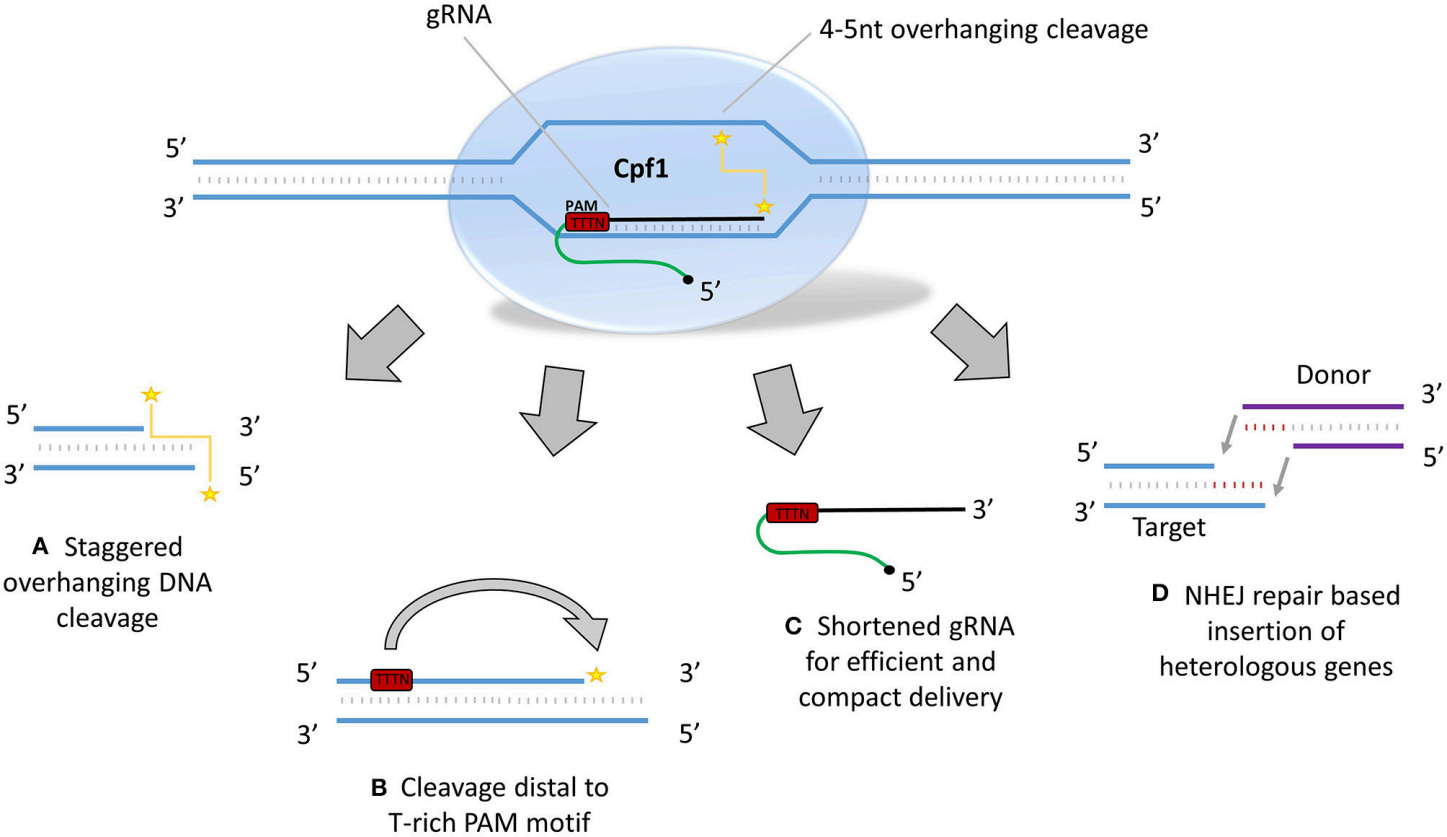
**Potentially beneficial features of Cpf1 vs. Cas9 for RGEN use in plants. (Top)** Cpf1-gRNA enzyme complex cleaving target DNA. Yellow staggered line with stars indicates overhanging DNA cleavage at sites distal to PAM. **(A)** Cpf1 creates staggered overhanging DNA cleavage where Cas9 creates blunt end DSBs. **(B)** DNA cleavage is distal to the thiamine-rich PAM recognition sequences opening up the prospect for enhanced HDR frequencies as recognition sites may not be abolished after NHEJ induced mutations distal to PAM. **(C)** Cpf1 gRNA is roughly half the size of Cas9, making delivery more compact and potentially efficient, especially for viral delivery methods. **(D)** Overhanging sticky ends after cleavage create the possibility for NHEJ mediated insertion of transgenes with directionality.

A major development in mammalian cell genome editing has been the report of a novel class II CRISPR RNA guided nuclease that cleaves DNA leaving 4-5-nt overhanging “sticky” ends as opposed to the blunt end digestion of Cas9 nucleases (Figure 3A). Cpf1 (CRISPR from *Prevotella* and *Francisella* 1) was developed based on reports of the type V CRISPR-Cas system found in various bacteria (including Primotella, Francisella, Acidaminococcus^*^, Lachnospiraceae^*^) and was shown to exhibit heterologous RGEN activity in mammalian cells (Zetsche et al., 2015; Yamano et al., 2016) (*only Cpf1 from *Acidaminococcus* and *Lachnospiraceae* exhibited heterologous nuclease activity in human cells). Cpf1 recognizes a thymine rich (TTTN) protospacer adjacent motif (PAM) sequence while Cas9 recognizes Guanine rich (NGG) PAMs. Cpf1 cleaves DNA distal to its PAM as opposed to Cas9 which cleaves DNA close to its PAM (Figure 3B). Because target sequences close to PAMs largely determine target specificity and nuclease activity for Cas9 (Jinek et al., 2012; Cong et al., 2013) and Cpf1 (Zetsche et al., 2015; Kim, D. et al., 2016; Kleinstiver et al., 2016), it is likely that Cpf1 mutated target sequences may be susceptible to repeated cleavage by a single gRNA, hence promoting Cpf1's application in HDR mediated genome editing. Moreover, this novel RGEN appears to lack two distinct active nuclease domains such as Cas9, but rather is a homodimer of an active RuvC-like domain that, when mutated to abolish activity (as deactivated Cpf1 or dCpf1), does not cut either strand of DNA substrate. Cpf1 does not require transactivating CRISPR RNA (tracrRNA) and thus gRNAs are shorter in length than those for Cas9 by about 50%, having great impact on delivery options, especially for viral mediated delivery (see above, Figure 3C). Further, Overhanging sticky ends resulting from Cpf1 cleavage can facilitate NHEJ mediated insertion of transgenes with directionality (Figure 3D). Cpf1 also seems capable of cleaving RNA (Fonfara et al., 2016), which potentially adds another important functionality to this nuclease class.

Recently, genome-wide targeting specificity of the Cpf1 system was comprehensively analyzed in human cells by two independent groups and both groups concluded Cpf1 is a highly specific nuclease system suitable for precise genome editing (Kim, D. et al., 2016; Kleinstiver et al., 2016). Two additional papers reported generation of mutant mice by CRISPR-Cpf1, either by RNA delivery (Kim, Y. et al., 2016) or ribonucleoprotein delivery (Hur et al., 2016). Cpf1 was also shown to function in *Drosophila* (Port and Bullock, 2016). A general trend with RGEN developments in plants has been the validation of novel technologies first discovered in mammalian cell systems. Should this trend continue, we predict to hear reports of Cpf1 function in plants shortly. Considering that Cpf1 cleaves DNA differently than Cas9, recognizes totally different PAM sequences and may enhance RGEN gene insertion strategies, we are eagerly awaiting its arrival and use in plant systems.

## Author contributions

LL and YQ conceived and wrote the draft. AM drafted Figure 2 and revised the draft. All authors wrote, read and approved the final manuscript.

### Conflict of interest statement

The authors declare that the research was conducted in the absence of any commercial or financial relationships that could be construed as a potential conflict of interest.
